# Nanopatterned Cell Sheet Assembly of Biomimetic Cardiac Laminae for Modeling Structure–Function Relationships

**DOI:** 10.34133/bmr.0339

**Published:** 2026-03-05

**Authors:** Alex Jiao, Jesse Macadangdang, Jinsung Kim, Charles Travis Moerk, Nathan J. Palpant, Paulos Y. Mengsteab, Hyeon-Cheol Park, Charles E. Murry, Deok-Ho Kim

**Affiliations:** ^1^ Department of Bioengineering, University of Washington, Seattle, WA, USA.; ^2^ Institute for Stem Cell and Regenerative Medicine, University of Washington, Seattle, WA, USA.; ^3^ Center for Cardiovascular Biology, University of Washington, Seattle, WA, USA.; ^4^ Department of Biomedical Engineering, Johns Hopkins University, Baltimore, MD, USA.; ^5^ Center for Microphysiological Systems, Johns Hopkins University, Baltimore, MD, USA.; ^6^ Department of Stem Cell Biology and Regenerative Medicine, Eli and Edythe Broad Center for Regenerative Medicine and Stem Cell Research, University of Southern California, Los Angeles, CA, USA.; ^7^ Division of Cardiology, Department of Medicine, Johns Hopkins University, Baltimore, MD, USA.

## Abstract

Replicating the intricate 3-dimensional architecture and coordinated function of native human myocardium remains a central challenge in cardiac tissue engineering. Here, we present a scaffold-free strategy to fabricate multilayered human cardiac tissues with tunable structural anisotropy and physiologically relevant helical alignment. By integrating biomimetic nanotopographical patterning with a thermoresponsive polymer interface, we generated aligned cardiac cell sheets that could be detached and transferred intact. To ensure robust sheet formation and release, our comprehensive investigation found that a coculture system incorporating human induced pluripotent stem cell-derived endocardial-like endothelial cells was essential for facilitating extracellular matrix deposition and maintaining tissue integrity during detachment, outperforming coculture conditions using other stromal cell types. A glycidyl methacrylate (GMA)-modified polyurethane acrylate substrate functionalized with poly(N-isopropylacrylamide) enabled temperature-controlled release, with 0.5% GMA yielding optimal performance. Stacked cardiac sheets with defined angular offsets were used to engineer 4-layered laminae that mimicked the transmural fiber orientation of the ventricular wall. These helically aligned tissues exhibited enhanced contractile synchrony and superior contractile function compared to unaligned or unpatterned controls, as quantified by vector-based contraction analysis. This work introduces a modular, bottom-up platform for constructing functionally anisotropic cardiac tissues, providing new tools for probing myocardial biomechanics, studying development and disease, and informing regenerative therapies.

## Introduction

The native human myocardium features a highly organized 3-dimensional (3D) architecture composed of transmural layers of anisotropically aligned myofibers [1,2]. These myofiber sheets, referred to as laminae, are stacked assemblies of myocytes 4 to 6 cells thick. Within each lamina, elongated cardiomyocytes are aligned with extracellular matrix (ECM) fibers [3]. Across the ventricular wall, laminar exhibit a gradual rotation in orientation from a right-handed helix in the subendocardium to a left-handed helix in the subepicardium [4,5]. This transmural helical pattern is essential for coordinating the heart’s electrophysiological and mechanical functions. The gradual rotation of fiber orientation across the ventricular wall strongly influences the direction-dependent spread of electrical impulses, supporting rapid longitudinal conduction along myofibers and controlled transverse conduction between laminae [6–8]. It also influences myocardial stress and strain distribution [9,10], and contributes to transmural gradients of coronary perfusion, oxygen consumption, and metabolic demand, thereby contributing to the heart’s regional perfusion profile [11]. Mechanically, the helical architecture underlies the characteristic systolic twisting and diastolic untwisting, or “wringing”, motion of the left ventricle [12–14], which enhances ejection efficiency and promotes rapid ventricular filling through elastic recoil [15,16]. Disruption of this structural hierarchy is a hallmark of pathological remodeling and is implicated in the progression of cardiomyopathies, including hypertrophic and dilated forms [17–19]. Taken together, these observations underscore that the laminar–helical organization of the ventricular wall is not merely an anatomical feature but a key determinant of anisotropic electrical conduction, ventricular torsion, and transmural perfusion and energetic demand.

Human induced pluripotent stem cell (hiPSC) technology enables the derivation of cardiomyocytes and other cardiac cell types from adult somatic cells for drug testing, disease modeling, and regenerative therapy. However, current cardiac tissue engineering approaches have yet to recapitulate the complexity of native myocardial architecture. Most engineered tissues remain limited to uniaxially aligned [20–22] or randomly organized cardiomyocytes [23,24], falling short of mimicking the structure–function relationships found in native myocardium. Top-down approaches using decellularized organ scaffolds offer anatomical fidelity but lack cellular density and precise control over local tissue architecture [25,26]. Bottom-up strategies have emerged to provide finer spatial control of cell alignment by leveraging micro- and nanoscale surface cues, such as patterned ECM proteins or nanotopographies, to direct cell organization in 2D [27–29]. While these platforms successfully generate monolayers of aligned cardiomyocytes, extending this alignment into thick, multicellular constructs remains challenging. The heart’s functional output is determined not only by single-layer alignment but also by the coordinated orientation of successive myocyte layers forming laminar and helical tissue units [4,9,30–32]. As a result, in vitro tissues that more faithfully reproduce laminar and helical architecture may offer improved platforms for studying conduction abnormalities, altered ventricular torsion, and regional perfusion defects that emerge when this hierarchy is disrupted in disease.

To address these limitations, we engineered a scaffold-free platform that enables the fabrication of multilayered constructs with programmable helical architecture. Building on our previously established nanofabricated substrates [3,33], we incorporated a thermoresponsive polymer layer to enable intact detachment of anisotropic cardiac cell sheets. To overcome the challenge of sheet integrity during release, we introduced a coculture strategy using hiPSC-derived endocardial-like endothelial cells (ECs), which facilitated ECM deposition and enhanced monolayer cohesion. By stacking these sheets with controlled angular offsets, we created 3D cardiac tissues that recapitulate aspects of the ventricular transmural helical structure. Using vector-based contraction analysis, we demonstrate that tissue-level structural organization substantially modulates contractile performance, revealing a direct link between engineered tissue architecture and function. In particular, this modular, laminar–helical design provides a bottom-up framework for systematically interrogating how programmable changes in transmural fiber orientation influence conduction patterns, tissue-level twist-like motion, and, in future studies, perfusion-related phenomena in engineered cardiac tissues. This platform provides a robust and reproducible strategy to study cardiac biomechanics and development while offering new avenues for the design of physiologically relevant cardiac grafts.

## Materials and Methods

### Thermoresponsive nanofabricated substratum fabrication

The thermoresponsive nanofabricated substrate was fabricated following an established protocol as previously reported [34]. Briefly, a polyurethane acrylate (PUA; Norland Optical Adhesive) and epoxy-containing glycidyl methacrylate (GMA; Sigma-Aldrich) solution was mixed and processed using capillary force lithography to fabricate nanotopographic substrates, as outlined previously [18]. After polymerization, the resulting substrates were immersed in a solution of amine-terminated poly(N-isopropylacrylamide) (PNIPAM; Mn: 2,500, Sigma-Aldrich) prepared in deionized water and left to react for 24 h on a rocker at ambient temperature. To adjust the PNIPAM grafting density, the proportion of GMA in the mixture was varied across 0.5%, 1%, 5%, 10%, 15%, and 25% (v/v).

### Derivation and differentiation of hiPSCs into cardiomyocytes and ECs

Informed consent was obtained from all patients in accordance with Institutional Review Board (IRB) regulations. Urine-derived cells were isolated and expanded from a single healthy male donor using an established protocol [35]. A polycistronic lentiviral construct carrying human Oct3/4, Sox2, Klf4, and c-Myc4 was employed to reprogram these somatic cells into iPSCs. The resulting hiPSC line exhibited a normal 46, XY karyotype and was subsequently used for lineage differentiation. For cardiomyocytes, a modified monolayer-guided differentiation approach was adapted from prior work [6]. Briefly, 1 d prior to induction, undifferentiated hiPSCs were cultured in mTeSR 1 medium (Stem Cell Technologies) supplemented with CHIR-99021 (Selleck). On the induction day, the cells were treated with RPMI 1640 medium containing B-27 supplement without insulin, activin A (R&D Systems), and Matrigel (BD Biosciences). After 18 h, the medium was replaced with fresh medium containing bone morphogenetic protein 4 (BMP4) (R&D Systems) and CHIR-99021. On day 3, cultures received cytokine-free RPMI 1640 supplemented with B-27 without insulin (ThermoFisher) and XAV-939 (Tocris Bioscience). On day 5, cells were maintained in cytokine-free RPMI 1640 with B-27 without insulin, and beginning on day 7 and every other day thereafter, RPMI 1640 with B-27 supplement containing insulin was used. Spontaneous contractions appeared around day 7, and cells were maintained for an additional week before being reseeded at lower density (100,000 cells/cm^2^) for purification by metabolic selection, following established procedures [36]. Cardiomyocytes were used between 28 and 35 d post-induction and consistently showed ≥90% cardiac troponin T (cTnT) positivity.

For EC differentiation, a comparable monolayer-directed protocol was employed, adapted from published methods [37]. Briefly, following initial cytokine exposure, the culture medium was replaced with StemPro supplemented with ascorbic acid, BMP4, basic fibroblast growth factor (bFGF), and vascular endothelial growth factor (VEGF) for 3 d. Cells were reseeded at day 5 and subsequently maintained in endothelial-specific medium (EGM) containing CHIR-99021, bFGF, and VEGF to promote endothelial specification. ECs were characterized on day 5 by fluorescence-activated cell sorting (FACS) for CD31 expression, achieving ≥85% CD31^+^ populations, and were maintained in endothelial-specific medium until use at day 14.

### Culture of cardiac and stromal cells on the thermoresponsive nanofabricated substratum for cardiac cell sheet engineering

Human bone marrow–derived stromal cell lines hs27a and hs5 (Lonza) were revived from frozen stocks and expanded following the supplier’s recommended protocols. Human dermal fibroblasts (hDFs) were obtained through a skin punch biopsy performed on the forearm of a healthy 52-year-old male volunteer. Endocardial-like ECs were differentiated and maintained as outlined in the previous section. For cardiac cell sheet preparation, hiPSC-derived cardiomyocytes and stromal cells were enzymatically detached using 0.25% trypsin/EDTA (Lonza), resuspended, and combined at stromal fractions of 10%, 20%, or 30%. The resulting mixtures were seeded onto fibronectin-coated thermoresponsive nanofabricated substratum (TNFS) (5 μg/cm^2^) at a density of 175,000 cells/cm^2^. Cells were maintained in a 1:1 mixture of RPMI 1640 supplemented with B-27 (Lonza) and EGM (Lonza) and cultured for 7 d post-seeding. For fluorescent prelabeling of selected cell populations, cells were incubated in serum-free medium containing 2 μM CellTracker Green or Red (ThermoFisher) for 30 min before seeding.

### Transfer and stacking of nanopatterned cardiac cell sheets using a gel-casting method

Because cardiac sheets begin to contract once released from the TNFS surface, a controlled transfer technique is needed to preserve morphology. To achieve this, a gel-casting approach adapted from a published protocol [34] was used to transfer and stack nanopatterned cardiac cell sheets. Briefly, cell-seeded TNFSs were incubated in room-temperature Dulbecco’s phosphate-buffered saline (DPBS) for 30 min to initiate sheet detachment. Before complete release, DPBS was aspirated and 7.5% w/v gelatin in culture media prewarmed to 37 °C (Sigma-Aldrich) was added to the TNFS. The substrate was then chilled at 4 °C for 15 min to solidify the gelatine and stabilize the partially detached sheet while preventing premature detachment or folding. The TNFS was then incubated at 28 °C for 1 h for complete detachment of the cardiac sheet. The gel-casted nanopatterned cardiac cell sheet was transferred to a new substrate such as a glass coverslip precoated with Matrigel and plasma-treated (100 W, 5 min), or stacked directly onto another cell-seeded TNFS. Constructs were incubated for 2 h at 28 °C to promote adhesion for stacking. The stacking sequence was repeated up to 4 times to generate 4-layer nanopatterned cardiac sheets, which were subsequently transferred to plasma-treated Matrigel-coated glass coverslips for continued culture. Four-layered cardiac tissues were configured in 3 configurations: uniaxially aligned (aligned), helically oriented with ~20° rotation between sheets (helical), or unpatterned controls.

### Immunostaining, imaging, and quantitative analysis of cardiac cell sheets

Cells were washed with PBS (Sigma) and fixed in 4% paraformaldehyde for 15 min at ambient temperature (22 °C). Following fixation, samples were washed again in PBS and subjected to permeabilization and blocking using a solution of 5% bovine serum albumin (BSA; Sigma) with 0.25% Triton X-100 (Sigma) for 1 h. For multilayered constructs, the permeabilization/blocking step was extended up to 4 h to ensure reagent penetration. Cells and engineered sheets were then incubated overnight at 4 °C with primary antibodies including α-sarcomeric actinin (1:200, Abcam), fibronectin (1:1,000, Abcam), and CD31 (1:20, Abcam) diluted in 1% BSA/PBS. On the following day, samples were washed and labeled with secondary antibodies (Alexa Fluor series, Invitrogen) together with Alexa Fluor 488-conjugated phalloidin (1:200, Invitrogen) for 1 h at 37 °C. Nuclear staining was performed using Hoechst 33342 (1:1,000, Sigma). After final PBS washes, samples were mounted in Vectashield (Vector Laboratories) and imaged using a Nikon A1 confocal system mounted on a Ti-E inverted microscope. Confocal imaging was supported by the Mike and Lynn Garvey Cell Imaging Laboratory at the Institute for Stem Cell and Regenerative Medicine, University of Washington. To assess cytoskeletal alignment quantitatively, images from 3 representative regions acquired at 60× magnification were analyzed using a custom MATLAB-based pixel gradient algorithm adapted from previously published methods [34,38]. Images were first smoothed with a Gaussian low-pass filter and convolved with horizontal and vertical Sobel operators to compute the gradient magnitude and local orientation angle for each pixel. Thresholding was then applied to isolate regions of interest, and gradient orientations were calculated relative to the *x* axis (0°). For quantitative visualization, each image was subdivided into a user-defined grid, and gradient angles within each grid square were circularly averaged to generate a mean local orientation vector. These pixel-level orientation vectors were subsequently binned to produce orientation histograms, from which the dominant alignment angle and angular dispersion were extracted. For multilayer aligned and helical constructs, confocal Z-stacks were segmented into lamina-specific regions of interest, and layer-specific orientation distributions were analyzed to quantify the angular offset between successive layers.

### Correlation-based contraction quantification analysis of cardiac cell sheet contractile function

To evaluate contractile performance, videos of electrically paced cardiac sheets were recorded. The culture medium was exchanged for prewarmed Tyrode’s solution, and tissues were stimulated using a MyoPacer Field Stimulator (Ionoptix) with 1-Hz frequency, 10-V amplitude, and 5-ms square-wave pulses. For each sample, 4 to 5 contraction cycles were captured across 3 to 5 independent fields of view. The recordings were analyzed using a correlation-based contraction quantification (CCQ) algorithm adapted from our prior work [39]. In brief, an initial frame was segmented into a grid of fixed size windows, which were then cross-correlated with subsequent frames to determine local displacements. Window displacements were assembled into a vector field from which contraction angle was computed and, after spatial averaging, contraction magnitude as well as contraction and relaxation velocities were obtained. The correlation function produced Gaussian-shaped peaks, enabling subpixel resolution in displacement measurements. The videos used to perform the analysis were taken at 60 frames per second.

### Statistical analysis

Comparisons between unpatterned controls and nanopatterned cardiac cell sheets were assessed primarily with 2-way analysis of variance (ANOVA), followed by Tukey’s post hoc testing for multiple comparisons, using SigmaPlot software unless otherwise indicated. For quantification of contraction angle distributions, chi-square testing at a 5% significance threshold was performed in MATLAB. In all analyses, statistical significance was defined as *P* < 0.05. Data are presented as mean ± standard error of the mean (SEM).

## Results

### Formation of anisotropic cardiac cell sheets on the TNFS requires standardized cardiomyocyte inputs and optimized surface chemistry

**Fig. 1.**
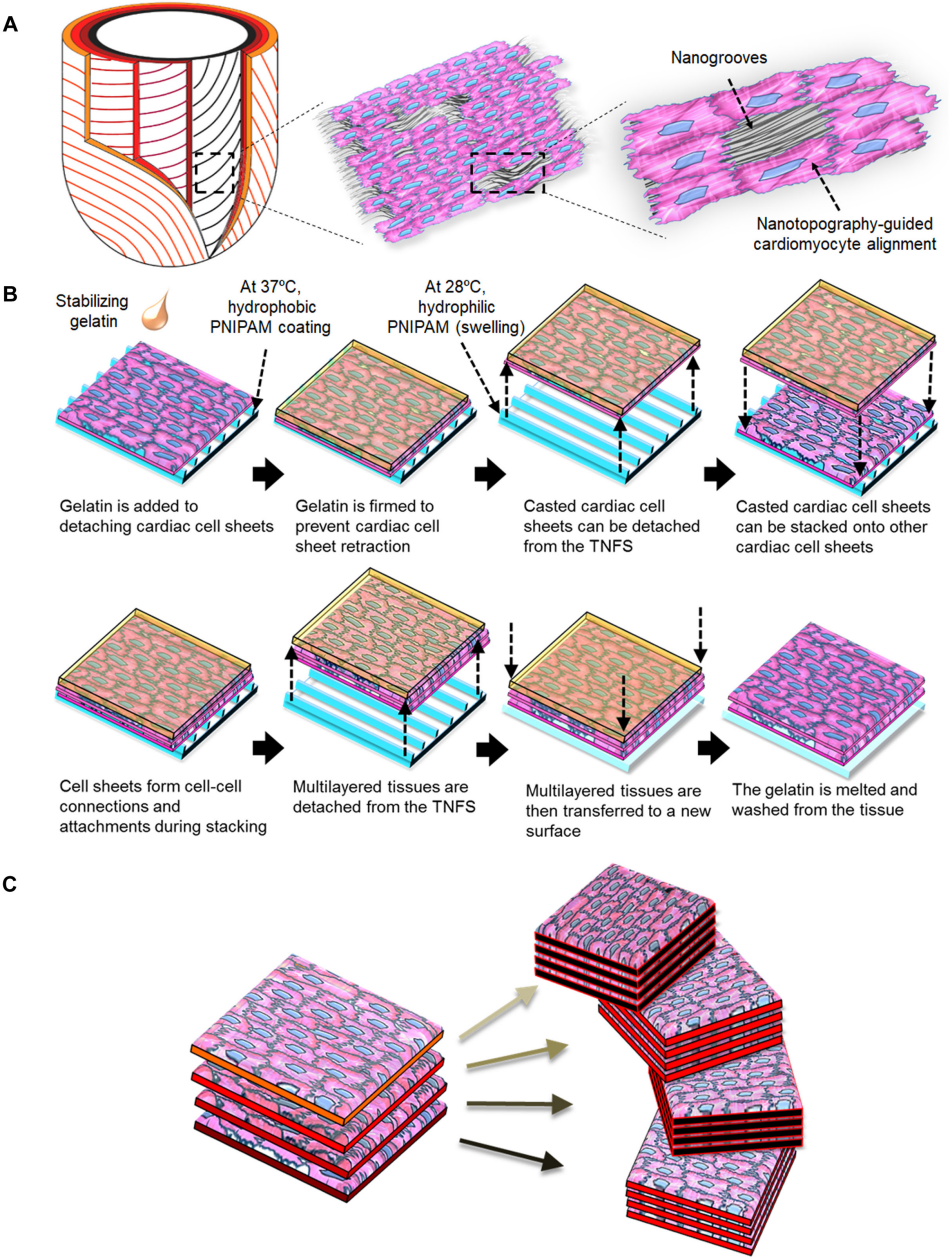
Bioinspired design of a thermoresponsive, nanostructured platform for engineering organized cardiac tissues. (A) Conceptual diagram of helically arranged myocardial layers in the native heart, consisting of anisotropic sheets of aligned cardiomyocytes and ECM fibers approximately 4 to 6 cells in thickness. This work provides a biomimetic approach to reconstruct such architecture by generating nanotopography-guided endocardial–cardiomyocyte coculture sheets on a thermoresponsive nanofabricated substratum (TNFS), where nanoscale grooves provide contact-guidance cues that direct cardiomyocyte alignment along the pattern axis. (B) Schematic illustrating the fabrication workflow using the TNFS. Nanoscale grooves on the TNFS provide contact-guidance cues that direct anisotropic alignment of endocardial–cardiomyocyte coculture sheets. During detachment, cooling below the PNIPAM phase-transition temperature hydrates and swells the thermoresponsive polymer layer, weakening cell–substrate adhesion (1 h at 28 °C). A gelatin casting step stabilizes the monolayer during this temperature-induced transition, enabling intact, alignment-preserving sheet release. The detached aligned sheets are then transferred and stacked with defined angular orientations to create multilayered cardiac laminae with programmable architecture. (C) Schematic illustration of stacked modular laminae assembled in a helical configuration to recapitulate complex myocardial architecture.

To establish reproducible 3D, anisotropic cardiac tissues, we first applied nanotopographical cues inspired by the aligned, native cardiac ECM fibers found in the myocardium (Fig. 1A). While differentiation of hiPSCs typically produces cardiomyocytes with high purity, batch-to-batch variability and residual noncardiomyocytes can compromise subsequent experiments. To minimize this variability and ensure consistent formation of cardiac cell sheets, we incorporated metabolic selection to purify cardiomyocytes in every differentiation run. Through metabolic selection, noncardiomyocytes were efficiently eliminated within the first few days, yielding highly purified, spontaneously beating cardiomyocytes that provided consistent input populations for sheet fabrication (Fig. S1 and Movies S1 and S2). With consistent cell sources established, we next optimized the surface chemistry of the TNFS to enable reliable formation of nanopatterned cardiac sheets. We systematically tested different concentrations of GMA (0.5%, 1%, 5%, 10%, 15%, and 25% v/v) used for PNIPAM grafting. The resulting variation in PNIPAM density was expected to regulate both attachment and detachment of cell sheets, since overly dense grafting produced surfaces that were too hydrophobic to support confluent monolayer formation. Purified cardiomyocytes (>99% cTnT^+^) were seeded on these TNFS conditions to evaluate their capacity to form aligned and stable cardiac monolayers. Cardiomyocytes successfully established aligned monolayers on TNFS prepared with 0.5%, 1%, and 5% GMA, whereas higher concentrations (10%, 15%, and 25% GMA) failed to support confluent sheet formation (Fig. S2). Among these conditions, cultures on 0.5% and 1% GMA TNFS developed cohesive, synchronously beating layers that exhibited pronounced cytoskeletal alignment and well-defined sarcomeric striations after 7 d of culture (Fig. S3). To evaluate the thermoresponsive release of cell sheets, TNFSs were then incubated with room-temperature DPBS. Under all tested conditions, however, the cells detached irregularly and formed aggregates rather than releasing as continuous intact sheets (Fig. S4).

### Incorporation of hiPSC-derived endocardial-like ECs enables the thermoresponsive detachment of nanopatterned cardiac cell sheets from the TNFS

**Fig. 2.**
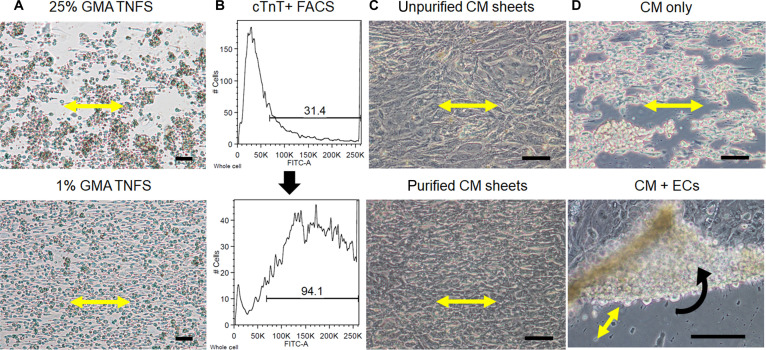
Substrate and cellular conditions influencing the formation of anisotropic cardiac sheets. (A) Representative brightfield images of TNFS fabricated with varying GMA concentrations, which modulate PNIPAM grafting density and thereby affect monolayer morphology. Scale bars, 100 μm. (B) Flow cytometric analysis of cTnT expression before and after metabolic selection, showing an approximately 3-fold enrichment of cardiomyocytes following purification. (C) Brightfield images of cardiac sheets generated from unpurified hiPSC-CMs (top) versus metabolically purified hiPSC-CMs (bottom) on 1% GMA TNFS, illustrating improved cellular alignment and syncytial monolayer organization when purified cardiomyocytes are used. Scale bars, 100 μm. (D) Brightfield images of nanopatterned cardiac sheets during detachment in the absence (top) or presence (bottom) of 20% EC coculture, indicating that stromal ECM support enables intact, spontaneous sheet release. Scale bars, 100 μm.

The successful release of intact cardiac sheets from thermoresponsive substrates requires sufficient ECM deposition by the seeded cells. Prior reports have established that, when cultures on PNIPAM-coated surfaces are cooled below the polymer’s lower critical solution temperature (32 °C), the hydrated polymer chains swell and weaken cell–substrate adhesion, allowing the ECM-rich layer and its associated cells to detach as a cohesive unit (Fig. 1B). During this process, cell–cell and cell–matrix junctions can be preserved, enabling the sheet to maintain its structural and functional integrity [40]. To identify candidate stromal cell types capable of supporting ECM deposition required for sheet detachment, we examined 4 different coculture conditions that have been used in previous cardiac tissue engineering studies: primary hDFs, stromal cell lines hs27a and hs5, and hiPSC-derived endocardial-like ECs. Each was combined with cardiomyocytes at varying ratios and cultured on TNFS substrates. Of these, hs5 cocultures consistently failed to support the development of stable monolayers (Fig. S5A). In contrast, hDFs, hs27a, and ECs produced confluent anisotropic layers of cardiomyocytes aligned along the underlying nanotopography (Fig. S5B to D). The ability of these cocultures to facilitate thermoresponsive release was then assessed by incubating cultures in room-temperature DPBS for 1 h. hDF cocultures remained firmly adherent under all tested conditions and additionally produced heterogeneous tissues characterized by patchy regions of cardiomyocytes beating out of synchrony (Fig. S6B and Movie S3). Sheets generated with hs27a or ECs detached readily from the substrate under the same conditions and were transferred using a gel-casting method. However, while hs27a cocultures yielded sheets that lost their structural alignment upon transfer (Fig. S6A), EC-cocultured tissues retained the anisotropic organization of the original monolayer (Fig. S6C). From these results, we determined that TNFS fabricated with 0.5% or 1% GMA and seeded at a 1:5 EC-to-cardiomyocyte ratio provided optimal conditions for generating cohesive, anisotropic sheets that detached spontaneously upon cooling (Fig. 2 and Movie S4). Importantly, the released sheets maintained cell–cell connectivity during the detachment process, as demonstrated by synchronously beating cardiac sheets after transfer (Movie S5). Although cultures maintained beating activity for up to 14 d, occasional premature detachment events were noted at the higher GMA concentration (1%). Consequently, subsequent transfer and stacking experiments were conducted using the 0.5% GMA TNFS condition as the more stable platform.

### Transferred nanopatterned cardiac cell sheets maintain alignment long-term and can be stacked to form multilayered, aligned cardiac tissues with discrete cardiac layers

**Fig. 3.**
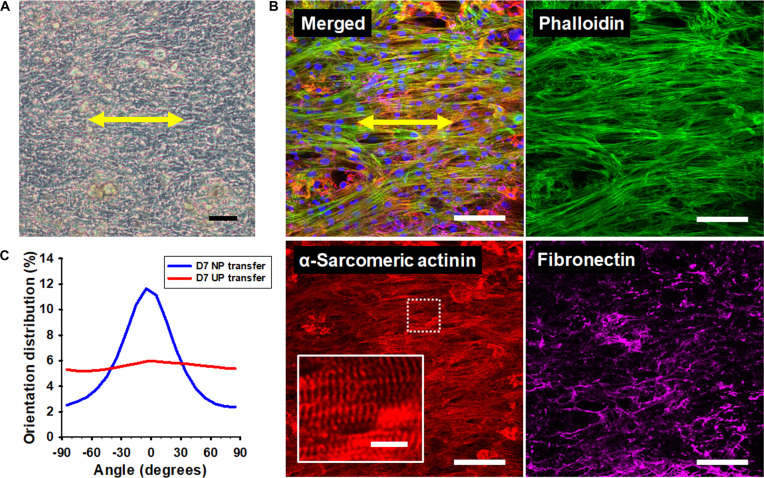
Nanopatterned endocardial–cardiomyocyte coculture sheets can be transferred to secondary surfaces while preserving alignment and matrix organization. (A) Brightfield image of an anisotropic cardiac sheet undergoing spontaneous detachment from a TNFS at 22 °C. Scale bar, 50 μm. (B) Confocal image of an immunostained cardiac sheet 7 d after transfer onto a glass coverslip, showing sustained cytoskeletal orientation, organized sarcomeric structures, and deposition of ECM proteins. Scale bar, 100 μm; inset, 10 μm. (C) Quantitative analysis of cytoskeletal orientation in nanopatterned (NP) cardiac sheets compared with unpatterned (UP) controls, highlighting enhanced alignment in NP sheets 7 d after transfer.

To assess the feasibility of engineering aligned cardiac tissues for further structure–function studies, we first evaluated the transfer of individual aligned cardiac sheets and subsequently extended this approach to stacked, multilayered tissues. Cardiac sheets generated on the nanopatterned substrates were transferred onto Matrigel-coated coverslips using the gel-casting approach. The transplanted sheets exhibited clear cytoskeletal alignment and rhythmic contractile activity immediately after relocation (Fig. 3A) and continued to display sustained contractility and structural alignment for at least 7 d in culture (Fig. 3B). Quantitative image analysis confirmed that the degree of alignment was significantly higher than in unpatterned controls (Fig. 3C). Within the transferred constructs, cardiomyocytes displayed organized sarcomeres and striated patterns, reflecting a more mature structural phenotype.

**Fig. 4.**
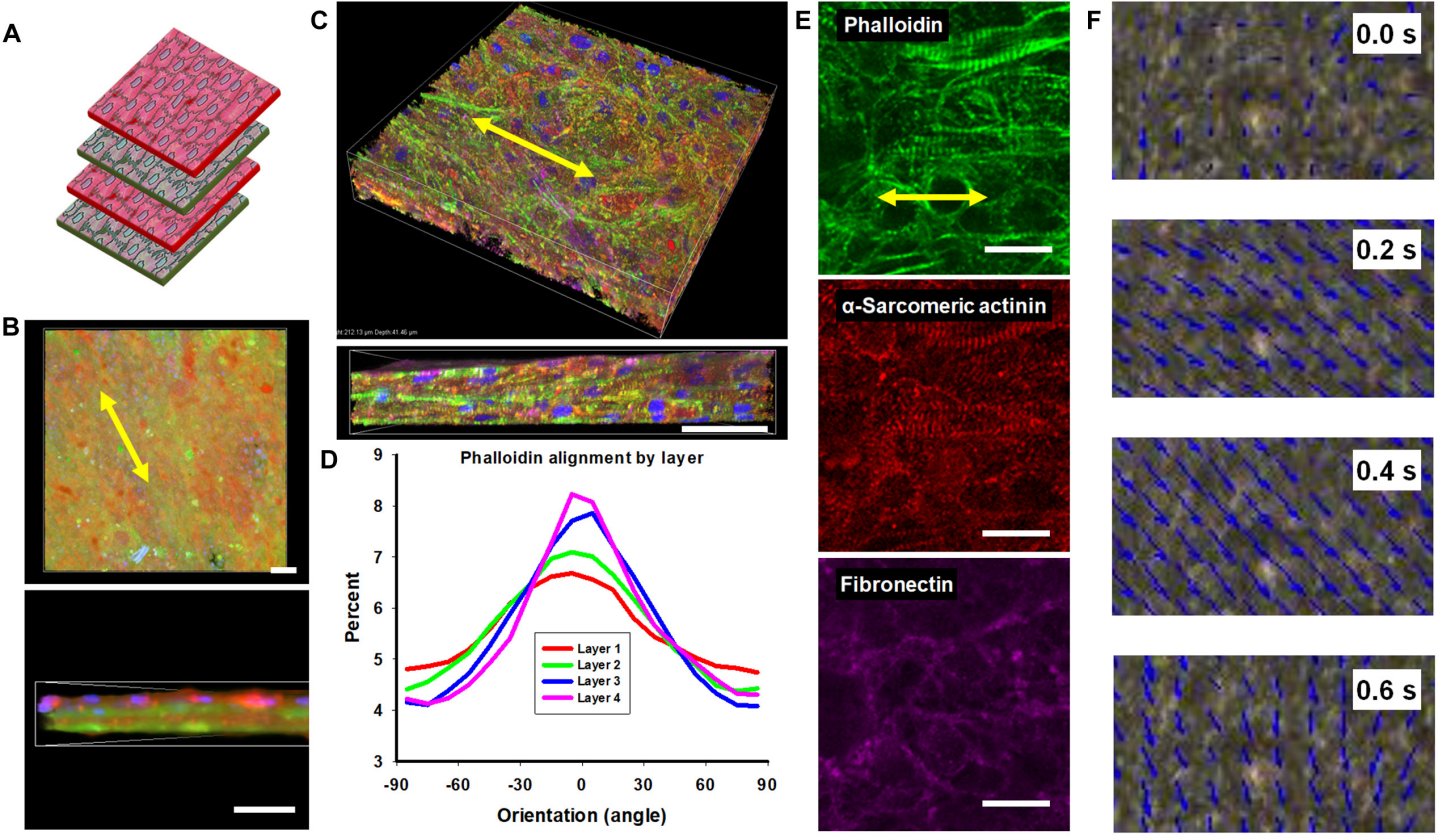
Aligned 4-layer cardiac tissues preserve laminar integrity and overall orientation. (A) Schematic illustrating fluorescently labeled cell sheets stacked in a red–green–red–green (RGRG) sequence with parallel alignment. (B) Confocal image of CellTracker-labeled cardiac tissue 1 d after stacking and transfer onto a glass coverslip, showing global orientation (top) and retention of distinct laminar layers (bottom). Scale bars: top, 100 μm; bottom, 40 μm. (C) 3D rendering of a confocal z-stack of immunostained tissues, highlighting aligned cytoskeletal organization (top) and compact, multilayered thickness (bottom). Scale bar, 40 μm. (D) Quantitative analysis of cytoskeletal orientation across all 4 layers, confirming persistence of uniaxial alignment both within individual sheets and across the composite tissue. (E) Representative immunostained single cardiac sheet exhibiting aligned cytoskeletal structure, ordered sarcomeres, and ECM deposition. (F) Sample frames from CCQ contractility analysis with vector overlays, illustrating coherent, unidirectional motion of aligned 4-layer tissues during contraction.

To fabricate multilayered engineered tissues, 4 individual cardiac sheets were sequentially stacked using the gel-casting technique, creating constructs with uniaxial alignment (aligned), helical alignment (helical), or unpatterned control tissues. These multilayer tissues were transferred onto Matrigel-coated surfaces, where they preserved their architecture and exhibited synchronous spontaneous contractions for at least 7 d in continuous culture, without obvious delamination or loss of interlaminar coupling, consistent with prior reports of long-term functional stability in scaffold-free cardiac sheet constructs [34,41]. Interestingly, during the initial stacking process, each lamina contracted independently and remained distinguishable as separate layers (Movie S6). After 24 h of culture, however, the layers exhibited synchronized contraction and relaxation, indicating progressive compaction and reinforced intersheet connectivity. To visualize the integrity of each lamina within the engineered constructs, cardiac sheets prelabeled with green or red dyes were alternately layered (Fig. 4A). When sheets were transferred after 7 d of culture, partial mixing between adjacent layers was observed, leading to heterogeneous interfaces (Fig. S7A). In contrast, when sheets were cultured for 14 d before transfer, discrete boundaries were preserved, and the individual layers maintained structural identity within the 4-layer assembly (Fig. 4B).

### Engineered multilayered cardiac tissues retain individual layer alignment even when stacked in complex 3D tissues, which subsequently affects tissue function

**Fig. 5.**
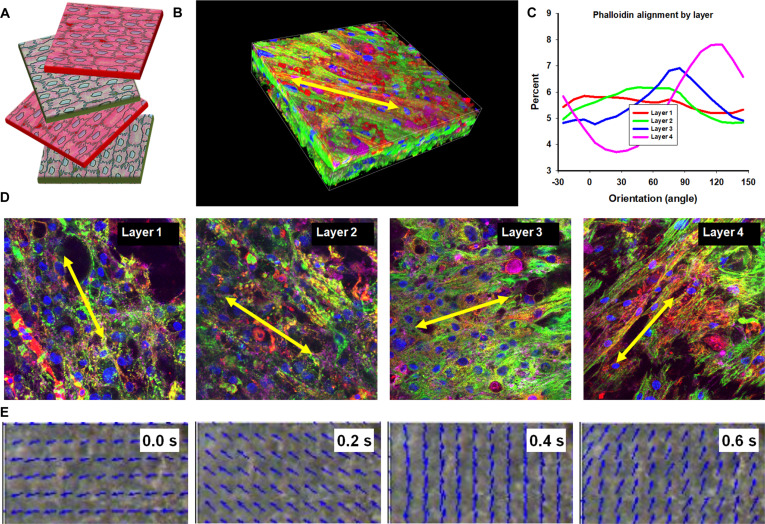
Helical, 4-layer cardiac tissues preserve laminar organization and exhibit rotation-like contractile motion. (A) Schematic illustration of a 4-layer construct assembled in a helical configuration. (B) 3D rendering of a confocal z-stack of immunostained helical tissue, highlighting both overall thickness and preserved layer architecture. Scale bar, 40 μm. (C) Quantitative analysis of cytoskeletal orientation across the stacked laminae, showing layer-specific angular distributions and a progressive rotation in dominant alignment between successive layers, consistent with the programmed helical offset. (D) Representative immunostained images of single layers within the helical construct, showing layer-specific cytoskeletal orientations. (E) Selected frames from CCQ functional analysis with vector overlays, illustrating the characteristic swirling motion of helically aligned 4-layer tissues during contraction.

To assess intra-tissue architecture, 4-layer cardiac tissues were immunofluorescently stained and imaged for cytoskeletal, cardiac, and ECM markers. Quantitative analysis indicated that alignment was preserved within each lamina, although the degree of alignment decreased from basal to apical layers (Figs. 4D and 5C). Organized sarcomeres, comparable to those in single nanopatterned sheets, were observed across the construct, and ECM deposition was evident (Fig. 4E). Confocal z-stacks were used to estimate a thickness of ~8 to 10 μm per layer, yielding a total laminae thickness of ~40 μm (Fig. 4C). No vascular structures were observed within the engineered tissues.

**Fig. 6.**
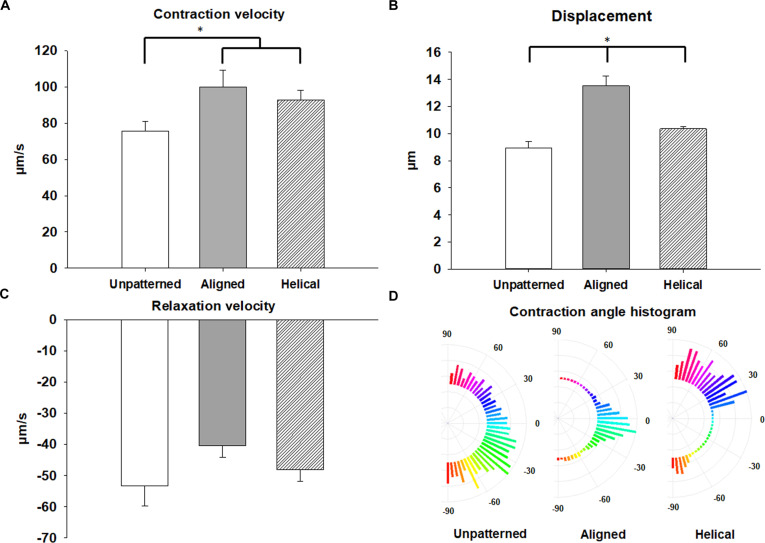
Structural organization enhances contractile performance in engineered cardiac tissues. Contractile dynamics were assessed using CCQ analysis of paced recordings from unpatterned, aligned, and helical 4-layer constructs. Quantification showed that aligned and helical tissues exhibited greater displacement (A), faster contraction velocity (B), and enhanced relaxation velocity (C) compared to unpatterned controls. (D) Polar plots of contraction angle distributions illustrate improved directional coherence in anisotropic tissues relative to controls.

CCQ-based analysis of contraction videos indicated predominantly unidirectional motion in aligned constructs and a swirling trajectory in helical constructs (Figs. 4F and 5E). To evaluate whether architecture influenced function, video recordings under paced field stimulation were analyzed for contraction magnitude, contraction velocity, relaxation velocity, and contraction angle dispersion. Compared with unpatterned controls, transferred aligned single sheets exhibited greater contraction magnitude, higher contraction and relaxation velocities, and reduced contraction angle dispersion (Fig. 6). Further increases in these endpoints were measured in multilayer aligned and helical tissues relative to single-sheet controls and multilayer unpatterned controls, with the largest improvements observed in aligned constructs.

## Discussion

In this study, we established a modular layer by layer strategy to build densely cellular cardiac constructs with programmable 3D architecture. Nanotopographic guidance mimicking myocardial ECM was used to generate uniformly aligned cardiomyocyte monolayers. A temperature responsive release combined with defined stromal coculture that promotes ECM accumulation enabled gentle intact sheet release and transfer while preserving alignment. Aligned sheets were subsequently stacked to yield multilayer constructs configured with customizable orientation across layers. Uniaxial and helical constructs were demonstrated as representative configurations in this study. After assembly, the constructs exhibited synchronous contractions across layers, while each lamina retained its anisotropic alignment, permitting construction of aligned and helical 3D cardiac tissues. Quantitative analysis showed architecture-dependent differences in contraction amplitude and in contraction and relaxation velocities, indicating that tissue-level orientation modulates functional output at the level of individual laminae. Although the heart contains an abundant amount of structurally organized ECM proteins, the myocardium is a cell-dense tissue. Cardiomyocytes must be in direct contact with one another to transmit an action potential and efficiently propagate electrical signals and generate force during systole [42,43]. As a result, the use of scaffolds to engineer cardiac tissue often limits functional outcomes by reducing native cell–cell interactions and inducing inflammatory responses, which in turn complicate integration with host myocardium [22,44]. The advent of scaffold-free cardiac tissue engineering has yielded promising results, specifically demonstrating improved cardiac function following transplantation. Nevertheless, to our knowledge, all previously reported scaffold-free cardiac constructs have not incorporated defined higher-order organization [45,46]. Our engineered, structured, 3D cardiac tissues demonstrated improved contractile properties over unstructured controls. For aligned 3D cardiac tissues, the coincident alignment of contraction direction and force vectors is expected to yield an additive effect. However, interestingly, even helically structured 3D cardiac tissues demonstrated improved contractile properties over controls despite the nearly perpendicular orientation of the top and bottom laminae. This likely reflects the preservation of anisotropic, uniaxial contraction within each lamina, in contrast to the isotropic contraction of unstructured controls. The anisotropic configuration may result in a greater overall contraction magnitude and velocity when summed throughout the 3D cardiac tissue, albeit blunted as the individual cardiac sheets were not aligned in a single direction. One notable observation was the progressive reduction in alignment across laminae from bottom to top. This is likely attributable to diminishing mechanical constraint, since the bottom layer was fixed to a rigid glass substrate, whereas the uppermost lamina rested only on adjacent cell sheets. This gradient in organization has several implications for tissue mechanics and structure–function coupling. Because individual cardiomyocytes develop maximal active tension along their long axis, highly aligned basal layers are expected to contribute disproportionately to directionally coherent force generation, whereas the more disorganized apical layers will produce force vectors with a broader angular distribution. At the tissue level, this partial loss of alignment in the upper laminae may therefore blunt the net contractile output along the principal fiber direction and increase shear components of strain within and between layers. In addition, reduced alignment is anticipated to broaden local stress distributions and partially decouple fiber-level shortening from coordinated wall thickening, which could, in more complex preparations, influence the efficiency of electromechanical coupling and the propagation of activation across stacked laminae. Although we did not directly measure transmural stress or electrical activation patterns in the present study, these findings highlight that maintaining high alignment throughout the tissue thickness is an important design parameter for maximizing contractile performance and physiological relevance. Future mechanical stress modeling may further elucidate the biophysical forces governing alignment maintenance in multilayer constructs. Additionally, a rigid top backing, such as the intra-laminae ECM in the heart, could be used to provide passive tension to maintain upper-layer alignment. However, despite the decrease in the degree of alignment observed in the topmost layer, overall 3D tissue structure still impacted contractile function, highlighting the structure–function relationship of cardiac tissue even within ~40-μm-thick tissue constructs. Considering that the human myocardium spans several millimeters [47], these engineered tissues could serve as promising building blocks for modeling larger myocardial segments and potentially whole-organ function.

The ability to engineer more complex 3D cardiac tissue structures, like helical architecture, provides an opportunity to more thoroughly investigate how cardiac architecture influences other disciplines, including developmental biology. For instance, although the adult cardiac structure is well-documented, the underlying processes regulating the development of this structure remain an area of ongoing investigation. Fibronectin and other ECM components facilitate the migration of early cardiac precursor cells during embryonic heart formation [48], followed by elongation of individual cardiomyocytes that establish lateral cell–matrix interactions with aligned ECM fibers [49] and then subsequent self-assembly of aligned fiber bundles into helical architectures during late fetal stages and continuing into postnatal remodeling [50]. These in vivo studies have suggested that both the ECM and the 3D cardiac microenvironment play roles in promoting cardiomyocyte maturation and shaping the myocardial architecture. Our platform could therefore serve as a tool to investigate how microenvironmental cues, including structural features, influence the differentiation and maturation of embryonic stem cell–derived cardiomyocytes. Subsequently, as we have used our platform with a variety of other cell types, including cells derived from similar developmental lineages as cardiomyocytes, it may also be adapted to generate multilayered cardiac tissues incorporating endocardium, myocardium, and epicardium, enabling systematic analysis of interactions between cardiomyocytes and supporting cells during development. Importantly, the modular laminar design also provides a scalable framework in which multiple aligned layers can be assembled into larger constructs while maintaining controlled fiber orientation and mechanical integrity. Because each sheet can interface with perfusable hydrogel scaffolds or microvascular channels, the platform is adaptable to thicker engineered tissues that require sustained metabolic support. Its planar configuration further enables seamless integration with soft bioelectronics for electrical stimulation, sensing, and real-time monitoring, offering a path toward functional cardiac grafts and physiologically responsive engineered tissues. We believe that this initial proof of concept demonstration of engineering diverse cardiac tissue architectures may yield valuable insights into stem cell biology and developmental processes while simultaneously advancing translational applications of cardiac tissue engineering.

## Data Availability

he data that support the findings of this study are available within the article and its Supplementary Materials files. Additional data and materials are available from the corresponding author upon reasonable request.
