# Supplementary material for: Nanopatterned Cell Sheet Assembly of Biomimetic Cardiac Laminae for Modeling Structure–Function Relationships
Source: Biomater Res. 2026 Mar 5;30:0339. doi: 10.34133/bmr.0339 (PMC12961156; doi:10.34133/bmr.0339)
Supplement: Supplementary 1 — Figs. S1 to S7 Movies S1 to S6 [file bmr.0339.f1.zip › BMR_Jiao_Supplementary.docx]

**SUPPLEMENTARY MATERIALS**


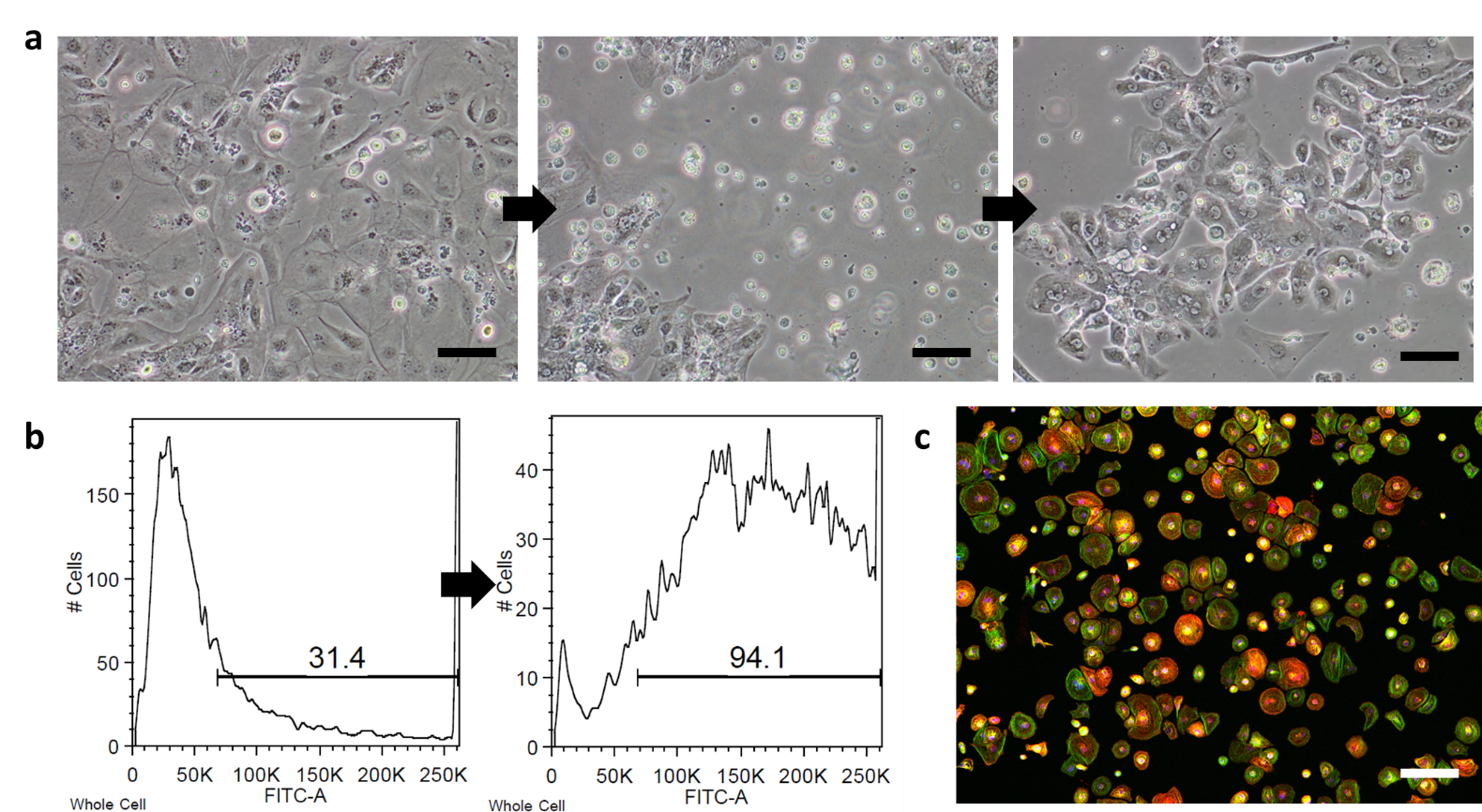


**Figure S1.** Metabolic purification of hiPSC-derived cardiomyocytes yields enriched cardiomyocyte populations. a, Microscope images over a 7-day culture period showing progressive loss of non-cardiomyocyte cells during metabolic purification. Scale bars, 100 µm. b, Flow cytometry analysis of cTnT+ cells before and after purification, demonstrating an approximately 3-fold increase in cardiomyocyte content. c, Immunofluorescence confirmation of purified cardiomyocytes stained for α-sarcomeric actinin (red), phalloidin (green), and Hoechst in blue. Scale bar, 200 µm.


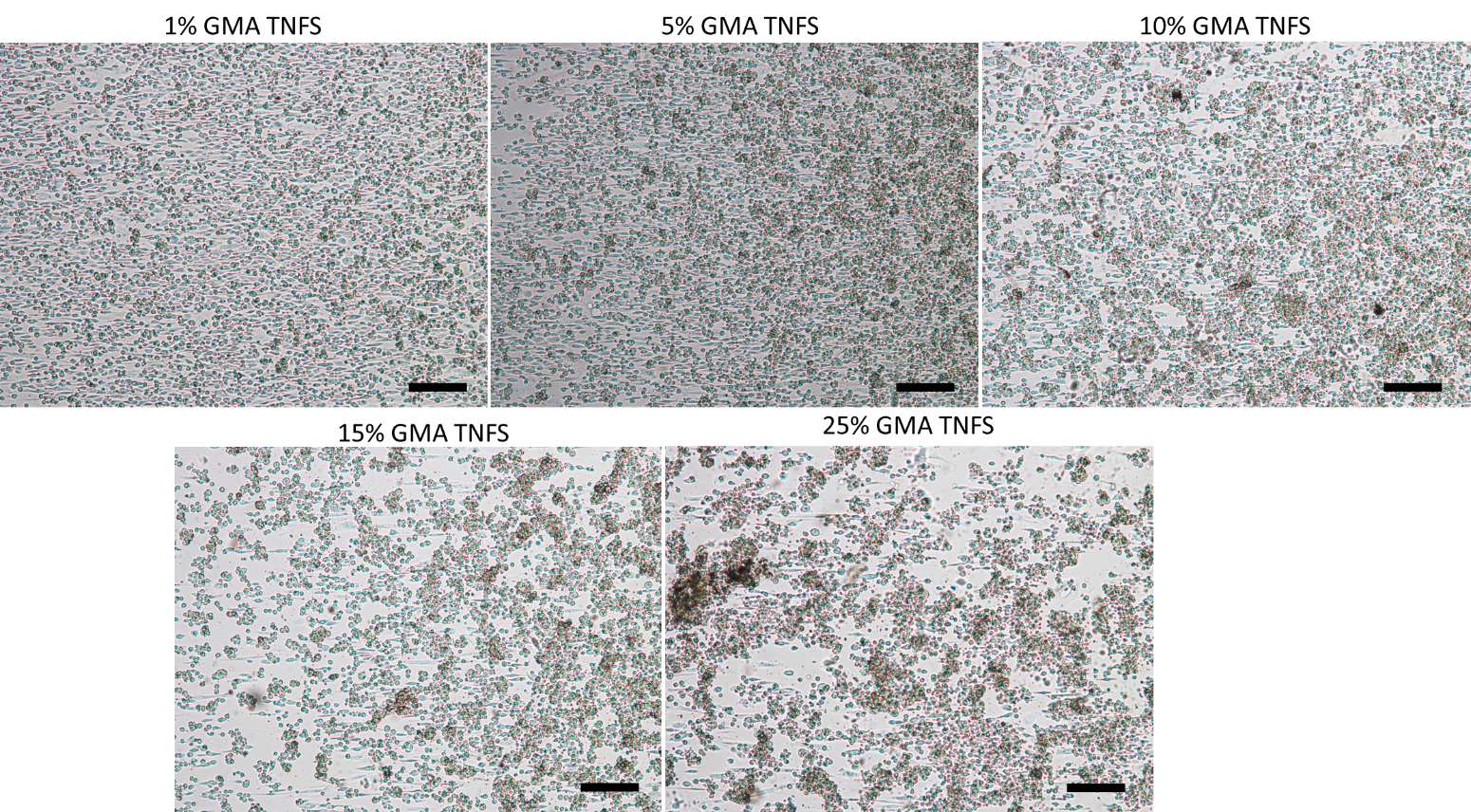


**Figure S2.** Effect of PNIPAM grafting density on cardiac sheet formation. Brightfield images showing how different GMA copolymer concentrations in the thermoresponsive nanostructured substrate (TNFS) influence monolayer organization and the ability to form anisotropic cardiac sheets. Scale bars, 200 µm.


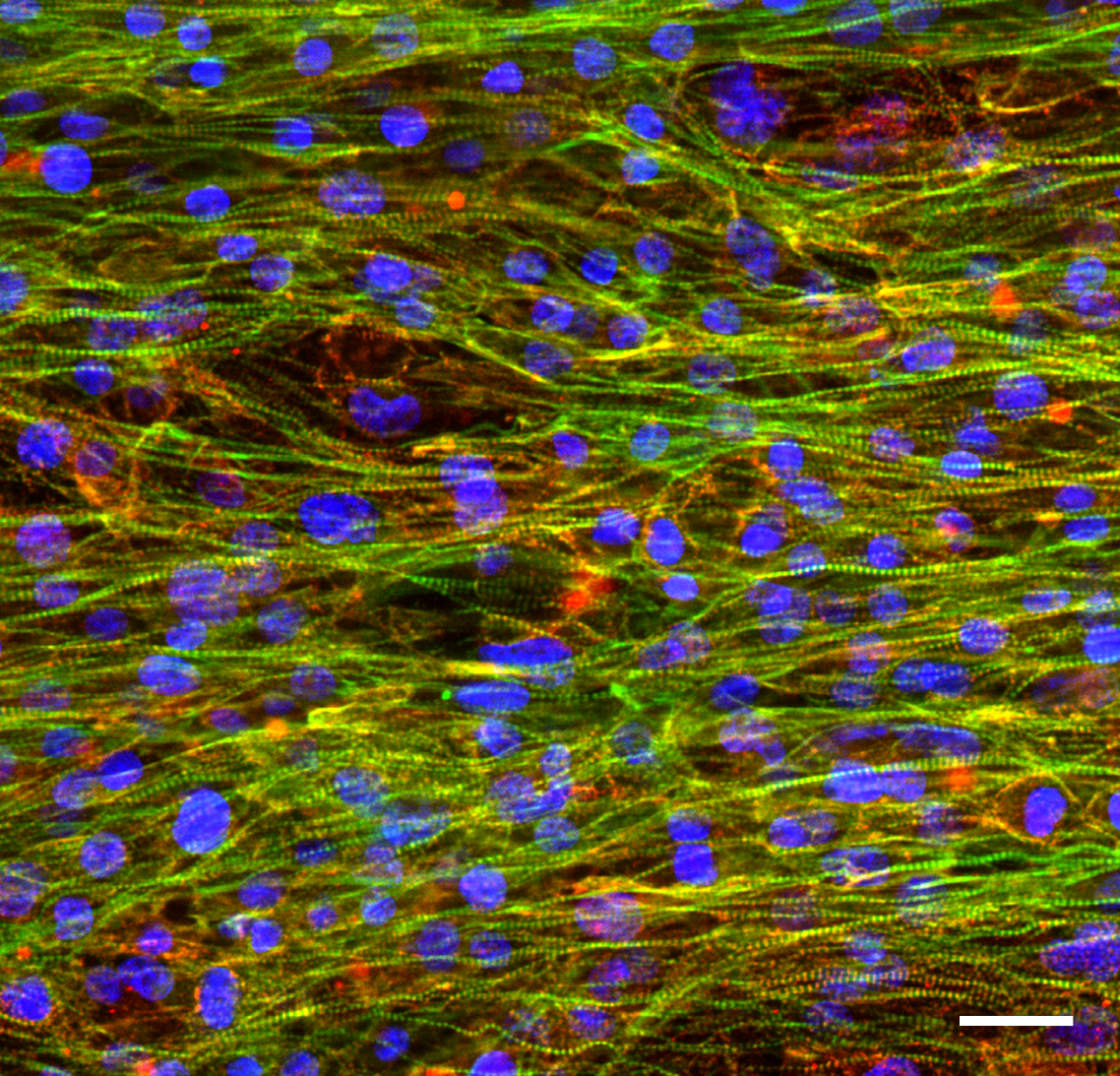


**Figure S3**. Alignment of structural proteins in pure cardiac cell sheets. Confocal immunofluorescence image of purified cardiomyocyte sheets cultured on TNFS, showing aligned sarcomeric structures. α-sarcomeric actinin (red), phalloidin (green), Hoechst (blue). Scale bar, 50 µm.


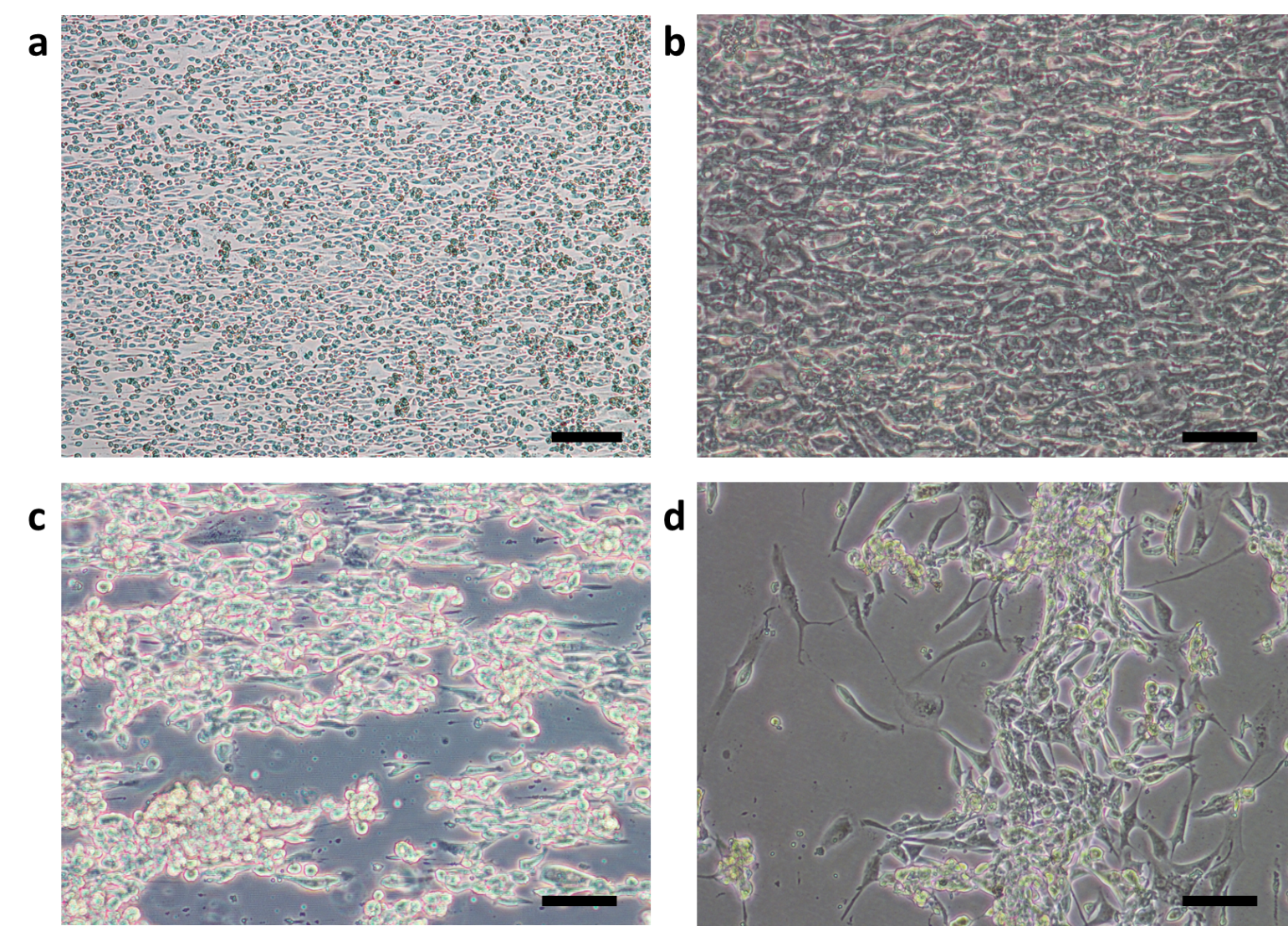


**Figure S4**. Pure cardiac cell sheets fail to detach as intact, anisotropic cell sheets. a, Purified cardiomyocytes (99% cTnT+) seeded on 1% GMA TNFS exhibit elongated and aligned morphology within 24 h. b, By day 7, cardiomyocytes form a syncytial, aligned monolayer. c, Temperature reduction from 37 °C to 22 °C triggers detachment from the surface, but not as an intact sheet. d, Transferred cardiomyocyte sheets lose initial alignment and show only partial transfer. Scale bars, 200 µm.


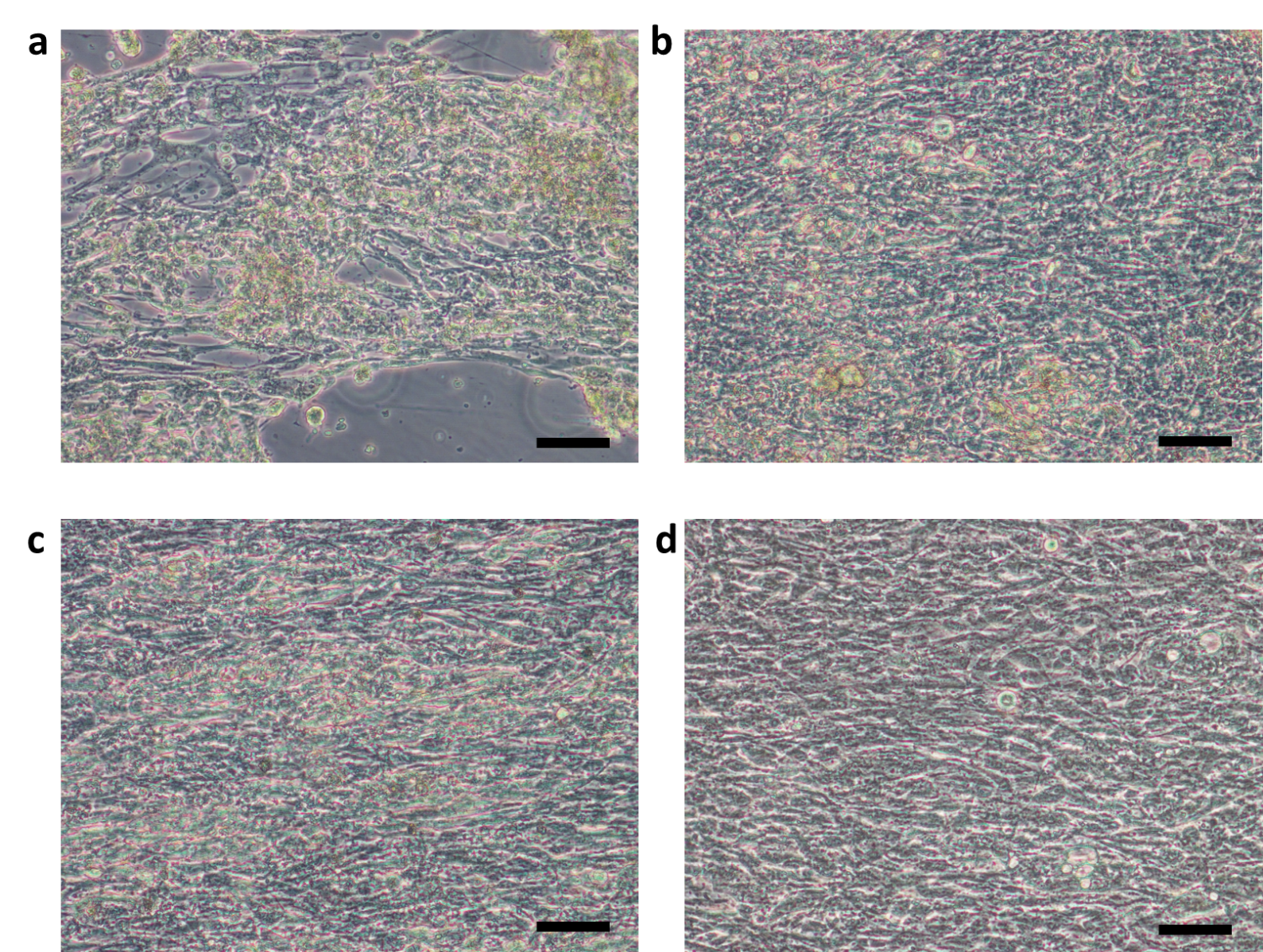


**Figure S5.** Stromal cell coculture with cardiomyocytes enables formation of detachable, anisotropic cardiac sheets. Brightfield images showing cardiomyocyte coculture with stromal cells: (a) hs5 stromal cells result in incomplete sheet formation, whereas (b) hs27a stromal cells, (c) primary human dermal fibroblasts, and (d) hiPSC-derived hemogenic anterior endothelial-like cells support aligned cardiac sheet formation. Stromal cells were mixed with cardiomyocytes at a 1:5 ratio. Scale bars, 100 µm.


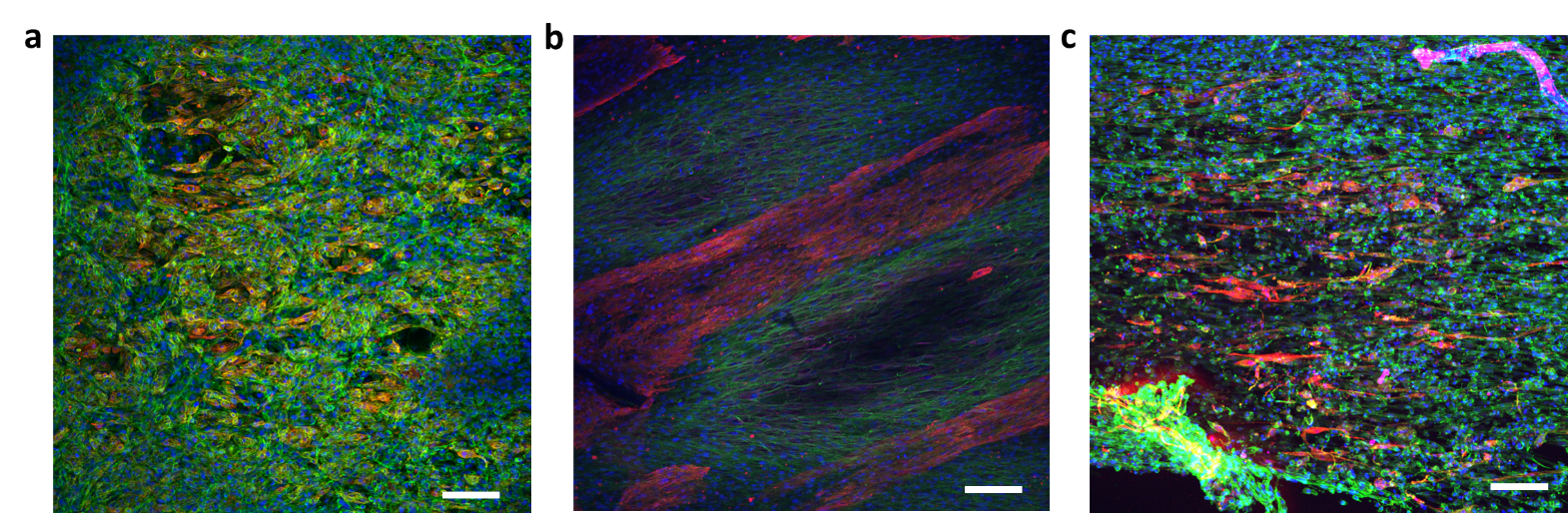


**Figure S6.** Endocardial cell coculture enables optimal formation and transfer of aligned cardiac cell sheets. a, Confocal microscope image of immunofluorescently stained hs27a cocultured cardiac sheets showing loss of alignment after transfer. b, Confocal microscope image of immunofluorescently stained hDF cocultured cardiac sheets demonstrating asyncytial beating with uneven cardiomyocyte distribution.

c, Confocal microscope image of immunofluorescently stained EC cocultured cardiac sheets forming well-aligned, syncytial monolayers. α-sarcomeric actinin in red, phalloidin in green, Hoechst in blue. Scale bars, 200 µm.


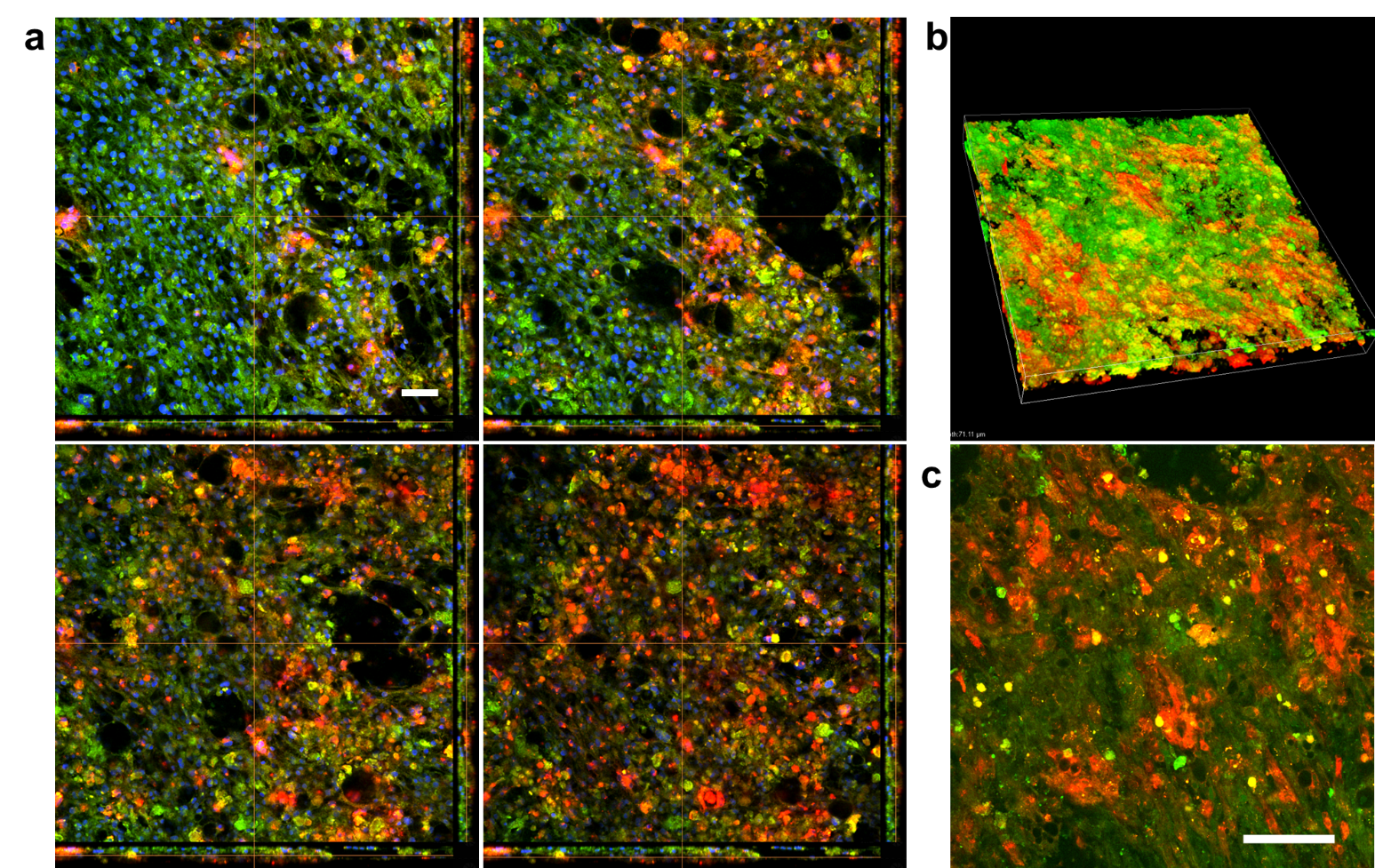


**Figure S7.** Engineered anisotropic cardiac sheets exhibit interlayer mixing and reorganization while maintaining alignment. a, Confocal microscope z-stack images of red and green membrane-labeled, 4-layer aligned cardiac sheets, showing progressive mixing between layers from bottom (upper left) to top (lower right). Scale bar, 100 µm. b, Confocal microscope z-stack 3D rendering of membrane-labeled cardiac sheets demonstrating intermixing of red and green cardiomyocytes. c, High-resolution confocal microscope image of a single layer showing intermixed red and green cardiomyocytes. Scale bar, 100 µm.

**Movie S1.** Representative video showing spontaneous beating of metabolically purified hiPSC-derived cardiomyocytes.

**Movie S2.** Representative video showing spontaneous beating of metabolically purified hiPSC-derived cardiomyocytes.

**Movie S3.** Representative video of cardiomyocyte cocultures with human dermal fibroblasts on TNFS showing patchy, out-of-sync beating associated with heterogeneous tissue organization.

**Movie S4.** Representative video showing spontaneous detachment of cohesive, anisotropic endocardial-like endothelial cell cocultured cardiac sheets from the TNFS upon cooling.

**Movie S5.** Representative video of a transferred cardiac sheet exhibiting synchronous beating after release and gel-casting transfer, indicating preservation of cell-cell connectivity.

**Movie S6.** Representative video captured during assembly of multilayer tissues showing independent contractions of each lamina immediately after stacking prior to interlayer synchronization.
